# Evolutionary Game Analysis of Behavior Strategies of Multiple Stakeholders in an Elderly Care Service System

**DOI:** 10.3390/ijerph20054263

**Published:** 2023-02-27

**Authors:** Zhiyong Zhang, Xiaodie Song, Yongqiang Shi

**Affiliations:** Department of Electronic Business, South China University of Technology, Guangzhou 510006, China

**Keywords:** elderly care services, regulatory strategies, evolutionary game, evolutionary stability strategy

## Abstract

As the aging of Chinese society continues to deepen, it is particularly important for the development of the national elderly care service industry to further strengthen the government’s supervision of private pension institutions and improve their management awareness of standardized operations. The strategic behaviors among the participants of senior care service regulation have not been well studied yet. In the process of senior care service regulation, there is a certain game association among three stakeholders, namely, government departments, private pension institutions, and the elderly. This paper firstly constructs an evolutionary game model including the above three subjects and analyzes the evolutionary path of strategic behaviors of each subject and the evolutionary stabilization strategy of the system. On this basis, the feasibility of the evolutionary stabilization strategy of the system is further verified through simulation experiments, and the effects of different initial conditions and key parameters on the evolutionary process and results are discussed. The research results show that (1) There are four ESSs in the pension service supervision system, and revenue is the decisive factor that affects the evolution of the stakeholders’ strategy. (2) The final evolution result of the system is not necessarily related to the initial strategy value of each agent, but the size of the initial strategy value will affect the rate of each agent’s evolution to a stable state. (3) The increase in the success rate of government regulation, subsidy coefficient and punishment coefficient, or the reduction in the cost of regulation and the fixed subsidy for the elderly can effectively promote the standardized operation of private pension institutions, but the large additional benefits will lead to their tendency to operate in violation of regulations. The research results can provide reference and a basis for government departments to formulate the regulation policy for elderly care institutions.

## 1. Introduction

At present, the aging of Chinese society has become one of the major social issues of general concern to the government and the public. Since China entered the aging society in 2000, the degree of population aging has deepened year by year. According to the statistics of the 7th National Population Census released by the National Bureau of Statistics, by the end of 2021, China’s elderly population aged 65 and above will be about 200 million, accounting for 14.2% of the total population of the country, which exceeds the standard of “deeply aging society” defined by the United Nations. This fully demonstrates the severity of the impact of the “silver wave” on Chinese society. The aging of the population has posed a huge challenge to China. In the face of such a huge demand for senior care services, it is not enough to rely on the government alone to provide them, so private pension institutions are operating [1]. Over the past two decades, the Chinese government has encouraged the development of private pension institutions by providing tax breaks and operating subsidies in response to the rapid growth in demand for senior care services [2]. Based on a series of preferential government policies, the number of private pension institutions in major Chinese cities continues to rise. According to relevant data, in Tianjin, China, there were only 4 government-run institutions in the 1980s, but by 2010, the number of institutions increased to 157 (including 20 government-run and 137 private institutions) [3].

It is well known that elderly people are a special group of people who often suffer from certain chronic diseases and may be severely disabled or semi-disabled [4]; this can lead to a low quality of elderly care services and increase the safety risks for elderly people if the monitoring system for elderly care services is not perfect, the personnel of institutions for the elderly are not professional enough, or there are safety hazards in the service process [5]. Therefore, in order to effectively protect the rights and interests of elderly people using elderly care services, the Chinese government has also introduced a series of policies to improve the elderly care service system in recent years, focusing on the need for the government to strengthen the supervision of the behavior of elderly care institutions, crack down on behaviors that infringe on the legitimate rights and interests of elderly people in elderly care institutions, and minimize the risks in elderly care services [6].

Although the state has promulgated a number of policy guidelines emphasizing the regulation of elderly care services, there are still many pain points in the actual implementation of regulation by lower government departments [7]. Under the market economy system, private pension institutions generally aim at maximizing their own interests [1], and in the face of the elderly, who are already vulnerable, they are more likely to commit illegal acts for their own interests and then violate the legitimate rights and interests of the elderly. According to the official news media in Sichuan, China, during the COVID-19 pandemic, illegal fund-raising of private elderly care institutions occurred frequently in Hunan, Guangdong, Zhejiang, Sichuan and other places, resulting in the loss of property and even life of many elderly people. By 2021, there were more than 6000 cases of illegal fund-raising by elderly care institutions for the people’s nutrition across the country. A total of 427 documents were found on the Chinese judicial documents website while searching with the keywords of “private pension institutions” and “illegal fund-raising” [8]. Therefore, the government needs to formulate a reasonable regulatory policy to protect the legitimate rights and interests of the elderly in using senior care services. However, the government’s decision to implement regulation will be affected by many factors, such as the cost of regulation and the success rate of regulation, so it is a question worth exploring whether the government is willing to implement regulation of senior care institutions. In addition, along with the revelations of negative news such as illegal fund-raising and caregiver injuries in some private pension institutions in recent years [2], the trust crisis between the elderly and private pension institutions has become more and more serious. These phenomena reflect the awareness that standardized operation of private nursing institutions still needs to be improved. For private pension institutions, there are also more factors that influence whether they choose to regulate their operations, such as operating costs, government policies on rewards and punishments, and so on. Therefore, whether or not private pension institutions choose to operate in a regulated manner is also an issue worth studying. In addition, the elderly are the core subjects of senior care services, and whether they choose to use senior care services or not also has an important impact on the strategy choice of the government and private pension institutions. For the elderly, whether or not to use senior care services is also influenced by the cost they pay and the benefits they receive, so whether the elderly are willing to choose to use private senior care services also needs to be explored. It can be seen that the providers, users and regulators of senior care services are all interconnected, and we can consider them as a complex and dynamic system. The interests of the provider and the regulator determine the quality of senior care services, and the involvement of the user also influences the strategy choices of the other two actors. Therefore, it is meaningful to consider the main stakeholders involved in the elderly care service and the dynamic process of their strategy choice in the elderly care service regulation model to accurately model the reality.

The issue of pension services has attracted the attention of many scholars. The existing research on elderly care services mainly focuses on the care service policy and qualitative analysis of the current situation [9,10], empirical research on the elderly care service system [11], and quantitative research on the elderly care service provision strategy [12]. However, they all ignore the multi-stakeholder interests involved in the provision of elderly care services. It is generally believed that coordination and cooperation among multiple subjects are the key factors to form a successful pension service system. Therefore, it is significant to include the main stakeholders in the elderly care service and their decision making and interaction process into studies. From the perspective of stakeholder research on elderly care services, the existing research focuses on the analysis of the relationship between elderly care institutions and government departments, with less consideration of the elderly as the core subject. At the same time, the existing research also pays less attention to the social reality that the illegal operation of private pension institutions frequently causes victimization of the innocent elderly. Therefore, this study will include the elderly as a group in the research scope and explore how government departments should coordinate the interests of the three parties and take regulatory measures to regulate the operation of private pension institutions, safeguard the legitimate rights and interests of the elderly, and achieve the long-term goal of promoting the healthy development of domestic elderly care services. Therefore, this paper establishes an evolutionary game model with multi-agent participation on the premise of considering the bounded rationality of all participants. Through the quantitative analysis of the model, we can obtain the evolution equilibrium of the pension service supervision system in different scenarios and the impact of different parameter changes on the behavior decisions of each subject. From the perspective of the system, dynamic decision-making analysis is carried out on the supervision of elderly care services, and the relevant management enlightenment is obtained, providing effective management suggestions for government departments to supervise private pension institutions and protect the rights and interests of the elderly.

The rest of the paper is structured as follows: in Section 2, the relevant literature on provision of elderly care services and evolutionary game theory (EGT) is reviewed. Section 3 introduces game relations among government departments, private pension institutions, and the elderly and describes model constructions and theoretical analysis. Section 4 simulates the ESS and external parameters with Matlab. A discussion and brief conclusions are given in Section 5 and Section 6.

## 2. Literature Review

### 2.1. Provision of Elderly Care Services

At present, many studies have been conducted by domestic and foreign scholars around the topic of elderly care services. The development of privatized senior care service institutions has attracted the attention of many scholars. In China, facing the rapid aging of the population, public elderly care institutions can no longer meet the huge demand gap for elderly care services, so the number of private pension institutions is on the rise year by year [7]. In addition, some scholars have also studied the privatization of elderly care in non-Asian countries. Maarse found through his research that certain European countries (such as Belgium, Poland, UK) are shifting from public to private in the provision of elderly care services [13]. Stolt et al. found through research that, faced with the pressure of population aging, the privatization of social pension services in Sweden has developed rapidly [14]. Although the rapid development of privatization of elderly care services has alleviated the shortage of elderly care service demand to a certain extent, it has also brought a series of problems and challenges. Taking China as an example, Feng et al. found through field research that some local government regulatory systems in China are weak, and government departments are faced with practical problems, such as the lack of pertinence of existing laws and regulations and the substandard quality of elderly care institutions’ facilities, making it difficult to ensure the quality and safety of elderly care services [6]. Relevant reviews also show that with the increasing participation of elderly nutrition departments in China’s long-term care system, the shortage of nursing staff and the weakness of quality assurance are becoming increasingly serious [7]. Therefore, in view of the many problems faced by the privatization of elderly care services, it is necessary for government departments to introduce reasonable policies to promote the healthy development of elderly care institutions.

Now, some scholars have shifted their research focus to elderly care service policy. Take China as an example: government departments have introduced operation subsidy policies for elderly care institutions. For example, in Beijing, the municipal government will provide a construction subsidy of USD 1270–2540 for each new bed and an operating subsidy of USD 16–32 for each bed in use [6]. In terms of elderly care service quality, Liu et al. found through field research that in Nanjing, China, the quality of elderly care services provided by public elderly care institutions is far higher than that of private pension institutions when there is no significant difference in elderly care facilities [2]. Hillmer et al. found through empirical research that for-profit elderly care institutions will provide lower quality nursing services in some aspects [15]. In addition, some scholars have explored other issues in the field of elderly care services. Tokunaga and Hashimoto use the Tobit regression method and find that the demand quantity and cost factors will significantly affect the market access options provided by for-profit elderly care services [16]. Through research, Kuhn and Nuscheler found that under the condition of information asymmetry, the nursing model of elderly care institutions is more popular than that of home care [17].

In summary, most of the existing studies use qualitative, empirical, and quantitative methods to study the elderly care service field from different perspectives, such as the privatization of elderly care services and the problems it brings, elderly care service policies, and the quality of elderly care services. However, they ignore the interaction analysis between the government, private pension institutions, and the elderly. Yue and Lin built an evolutionary game model of interactions between the government and private pension institutions to explore the impact of government punishment and operating subsidies on their evolutionary stability strategies [18]. He et al. built a game model of interactions between the government and the private sector to explore the evolution of the behavior strategies of both parties in the provision of elderly care services under different scenarios [1]. However, their analysis focuses on the behavioral strategies between government departments and private pension institutions, and less on the elderly as the core participants. In order to promote the utilization rate of elderly care services, Xi’an City in China has put forward a subsidy mechanism to provide funds for those who choose institutional elderly care. With the expansion of the demand market for elderly care services, the proportion of elderly people participating in the use of elderly care services will greatly affect the behavior decisions of government departments and elderly care institutions. Therefore, it is necessary for us to include the elderly group in the research on the institutional elderly care service system.

### 2.2. Evolutionary Game Theory and Its Application

The game theory method can provide a perfect mathematical framework for the analysis and prediction of the behavior of participants [19], which greatly improves the decision-making ability of each participant in the game system [20]. In the decision making on complex social issues, players in the game system often show limited rationality, and the information they hold is not completely transparent, so their decisions are not always correct [21]. Pension service supervision is a complex social problem. In the process of pension service supervision, the strategy choice of the government, private pension institutions, and the elderly will be affected by many factors. In addition, the existence of information asymmetry makes it difficult for the three parties to make correct decisions quickly. Therefore, a static game is no longer suitable for analyzing the dynamics and complexity of pension service supervision. EGT is different from static game theory; it emphasizes a dynamic equilibrium [22]. It does not require participants to be completely rational, nor does it require complete information [23]. From the perspective of system theory, EGT regards the adjustment process of group behavior as a dynamic system and can incorporate various factors that affect the behavior of the subject into the game model, so it can more truly reflect the diversity and complexity of the subject’s behavior. Therefore, EGT is more suitable for studying the complex social problem of pension service supervision.

In recent years, more and more scholars have used EGT to study problems in economics, social science, and other fields [24,25,26,27]. Based on EGT, Liu et al. built a game model between household medical device enterprises and the government and studied the behavior strategy changes of household medical device enterprises under the government’s dynamic reward and punishment mechanism [28]. Chen et al. explored how to guide the standardization of behavior decision making of the main body to control problems such as false publicity and insufficient after-sales service in live broadcast platforms by building an evolutionary game model of the government, consumers, and the live broadcast platform [29]. Li et al. focused on exploring the impact of public participation on government regulatory behavior in PPP projects based on EGT [19]. He and Qin take government regulatory departments and coal mining enterprises as research objects and obtain methods that are helpful for coal mine safety supervision by establishing and analyzing a two-party evolutionary game model [30]. Based on the current situation of incomplete development of construction waste industrialization, Li et al. established a game model of recycling units with and without remanufacturing capacity to explore the impact of government regulation rates and cost subsidy rates on the evolution behavior of recycling units [31]. In addition, some scholars have focused on how to combat the loss of biodiversity by building an evolutionary game model between humans and animals [32]. All of the above studies show that EGT is an effective tool for analyzing complex and dynamic social problems. In summary, EGT is applicable to the research on the complex social problem of pension service supervision. As far as we know, EGT has not been used to conduct relevant research on the game relationships among government departments, private pension institutions, and the elderly. Therefore, this paper will establish an evolutionary game model including government departments, private pension institutions, and the elderly to further explore the behavior strategies of each subject under different scenarios.

## 3. Model

Firstly, we describe the decision-making problem with the current situation of China’s pension service market. In recent years, the number of private pension institutions has increased sharply in the context of the sharp increase in domestic demand for pension services and the shortage of resources in public pension institutions [3]. The government has also issued a series of welfare policies, including tax reductions and construction subsidies, to encourage the rapid development of private pension institutions [2]. However, in view of the key characteristics of private pension institutions, which are profit-oriented, although the government has issued many policies and measures to strengthen the supervision of pension institutions, there are still many problems in the actual operation of pension institutions [7]. For example, in 2017, there were more than 30,000 service cases of elderly care institutions in Beijing, and 109 elderly care institutions failed to meet the standards in terms of infrastructure construction, catering qualification, and other indicators. In addition, with the exposure of scandals in recent years, such as illegal fund-raising of elderly care institutions, bullying of elderly care workers, and abuse of the elderly, the service quality of elderly care institutions is even more worrying. Therefore, in order to ensure the standardized operation of elderly care institutions and effectively protect the legitimate rights and interests of the elderly, it is necessary for government departments to carry out macro control. In the past decade, the domestic pension service system framework has been continuously improved, and pension service policy documents have been intensively issued. For example, in 2012, the state formulated the Basic Code for Elderly Care Institutions, which formulated the personnel requirements, management requirements, service content requirements, and environment and facility requirements for elderly care institutions to standardize the daily operation and management of elderly care institutions. At the same time, with the support of national policies, domestic cities have gradually introduced implementation rules to promote the development of local elderly care institutions, emphasizing funding standards for the standardized operation of elderly care institutions and punishment measures for illegal operation. For example, since 2019, in Shanghai, China, if elderly care institutions add new beds, the district’s finance will provide a start-up subsidy of CNY 5000 per bed. Elderly care institutions that violate the regulations on standardized operation will be warned or fined according to the seriousness of the case.

Based on the above analysis of the actual situation, it can be seen that the supervision of pension institutions by government departments is a long-term dynamic process. As the elderly group is also a key participant in the pension service supervision system, the government must ensure that the legitimate rights and interests of the elderly to participate in the use of pension services are not infringed. Therefore, this paper has developed an evolutionary game model among government departments, private pension institutions, and the elderly to further explore the interest relationships between the subjects and the impact of different parameters on the behavior decisions of each subject, and to find effective countermeasures to improve the regulatory capacity of government departments. The specific description and assumptions of the model are as follows.

This paper assumes that the three groups in the game are government departments, private pension institutions, and the elderly, all of which are limited rational participants. In the evolutionary game model, all players in the game are in the initial stage, and the strategies of all players in the initial stage are not optimal, so they need to continue to learn and adjust until they reach a balanced and stable state. In this process, other subjects that may have an impact on the players are not considered. The government departments have two strategic choices: One is to regulate the private pension institutions ($G_{1}$) to minimize the occurrence of irregularities in private pension institutions. The other is to choose not to regulate ($G_{2}$); that is, the government department will need to spend high human and material resources to implement supervision and choose to give up regulation. Private pension institutions have two strategic choices: standardized operation ($P_{1}$) and illegal operation ($P_{2}$). Private pension institutions choose standardized management strategies to provide high-quality elderly care services. The illegal operation refers to the illegal behavior of the private pension institutions in order to seek more benefits. The elderly have two strategic choices: participation ($E_{1}$) and non-participation ($E_{2}$). The elderly choose the participation strategy; that is, they use the services provided by private pension institutions. If they do not participate, they do not use the elderly care services.

### 3.1. Basic Assumptions and Model Parameters

Based on the analysis of the dynamic game relationship among stakeholders, we propose the following four specific assumptions:

(1) When government departments choose the $G_{1}$ strategy, they need to input fixed costs $c_{1}$($c_{1}>0$), including human, material, and financial resources in the regulatory process. At the same time, government department supervision will earn a reputation income from the public and a reward income from superior departments, so the total income obtained by the government departments by adopting the $G_{1}$ strategy is recorded as $\pi_{1}$ ($\pi_{1}>0$ and $\pi_{1}>c_{1}$). If the government departments do not supervise, it will make the public feel that the government departments do not act, thus causing damage to the reputation of the government departments. At the same time, government departments will also be subject to accountability and punishment from superior departments due to inadequate supervision. Therefore, the total loss incurred by government departments in adopting $G_{2}$ strategy will be recorded as $c_{2}$ ($c_{2}>0$). It should be emphasized that the government departments have a certain regulatory success rate when adopting the $G_{1}$ strategy, which is recorded here as *λ*, *λ* ∈ [0,1].
(2) When private pension institutions choose the $P_{1}$ strategy, they need to incur many infrastructure costs and higher labor costs, for which they pay a total cost $c_{3}$ ($c_{3}>0$) and obtain basic operating income $\pi_{2}$ ($\pi_{2}>0$). At the same time, the private pension institutions can receive a subsidy *s*(*s* > 0) from the government departments, and the subsidy coefficient is *θ*, *θ* ∈ [0,1]; that is, the subsidy amount is *θs*. When the private pension institutions choose the $P_{2}$ strategy, their input cost is $c_{4}$ ($c_{4}>0$), and at this time, the private pension institutions will receive some extra income Δ*π* (Δ*π* > 0) in some illegal ways, in addition to the basic operation income $\pi_{2}$. At this time, the private pension institutions will receive the punishment *p* (*p* > 0) from the government departments. The punishment coefficient is *η*, *η* ∈ [0,1], and the penalty amount is *ηp*, while pension institutions will speculate when operating in violation of regulations to reduce operating costs to obtain maximum benefits, $c_{3}>c_{4}$.
(3) When the elderly choose the $E_{1}$ strategy, they need to pay a certain service fee $c_{5}$ ($c_{5}>0$). In the process of using the elderly care service, they have gained the benefits of a happy mood, quality of life and happiness in life, which are recorded as $\pi_{3}$ ($\pi_{3}>0$). Considering the practical factors, it is assumed that the benefits of elderly care services are higher than their costs; that is, $\pi_{3}>c_{5}$. In addition, because the elderly choose to use the services provided by the private pension institutions, which is conducive to the development of the national elderly care industry, the government departments give a subsidy *a* (*a* > 0) to the elderly who choose the $E_{1}$ strategy. There is no cost to the elderly when they choose the non-participation strategy. As the elderly are the main consumers of elderly care services, if the elderly choose the $E_{2}$ strategy, it will bring operational losses to the private pension institutions. When private pension institutions choose the $P_{1}$ strategy and the elderly choose the $E_{2}$ strategy, it will bring a loss $c_{6}(c_{6}>0)$ to the private pension institutions. When private pension institutions choose the $P_{2}$ strategy and the elderly choose $E_{2}$ strategy, it will bring a loss $c_{7}(c_{7}>0)$ to private pension institutions. As private pension institutions invest more resource costs in standardized operations, if no one uses elderly care services, it will cause greater operating losses, so $c_{6}>c_{7}$. In addition, when private pension institutions operate in violation of the law, the elderly who participate in elderly care services will suffer a loss of benefits, recorded as $c_{8}$ ($c_{8}>0$).
(4) The government departments, private pension institutions, and the elderly will choose strategies according to their own wishes. Assuming that the probability of government departments’ choosing the $G_{1}$ strategy is *x*, the probability of government departments’ choosing the $G_{2}$ strategy is (1−*x*), *x* ∈ [0,1]. The probability of private pension institutions’ choosing the $P_{1}$ strategy is *y*, and the probability of choosing the $P_{2}$ strategy is (1−*y*), *y* ∈ [0,1].The probability of the elderly’s choosing the $E_{1}$ strategy is *z*, and the probability of choosing the $E_{2}$ strategy is (1−*z*), *z* ∈ [0,1].

The parameters in the model and their definitions are shown in Table 1 below.

### 3.2. Construction of the Model

Government departments, private pension institutions, and the elderly will choose relevant strategies according to their own wishes. Based on the above assumptions, this paper constructs the evolutionary game income matrix between government departments, private pension institutions, and the elderly as Table 2.

According to the game income matrix of government departments, private pension institutions, and the elderly constructed above, further calculate the expected income and average income of each subject under different strategies, and then construct their respective replication dynamic equations. 

Assume that the expected return when the government departments select the “regulation” strategy is $U_{g1}$, the expected return of choosing the “non-regulation” strategy is $U_{g2}$, and the average expected return is ${\bar{U}}_{g}$; according to the income matrix of the tripartite evolutionary game constructed above, it is calculated that:

(1) $$\begin{array}{c} U_{g1}=yz(\pi_{1}-c_{1}-\theta s-a)+y(1-z)(\pi_{1}-c_{1}-\theta s)+(1-y)z(\pi_{1}-c_{1}+\lambda \eta p-a)\\ +(1-y)(1-z)(\pi_{1}-c_{1}+\lambda \eta p)=y(-\theta s-\lambda \eta p)-za+\pi_{1}-c_{1}+\lambda \eta p \end{array}$$

(2) $$U_{g2}=yz(-c_{2})+y(1-z)(-c_{2})+(1-y)z(-c_{2})+(1-y)(1-z)(-c_{2})=-c_{2}$$

(3) $${\bar{U}}_{g}=xU_{g1}+(1-x)U_{g2}$$

According to the Malthusian dynamic equation [33], the replication dynamic equation of the government departments is:

(4) $$F(x)=\frac{dx}{dt}=x(U_{g1}-{\bar{U}}_{g})=x(1-x)[y(-\theta s-\lambda \eta p)-za+\pi_{1}+\lambda \eta p+c_{2}-c_{1}]$$

In the same way, the replication dynamic equations of private pension institutions and the elderly are as follows:

(5) $$F(y)=\frac{dy}{dt}=y(U_{p1}-{\bar{U}}_{p})=y(1-y)[x(\theta s+\lambda \Delta \pi +\lambda \eta p)+z(c_{6}-c_{7})-\Delta \pi +c_{4}+c_{7}-c_{3}-c_{6}]$$

(6) $$F(z)=\frac{dz}{dt}=z(U_{e1}-{\bar{U}}_{e})=z(1-z)(xa+yc_{8}+\pi_{3}-c_{5}-c_{8})$$

According to Equation (4), it can be derived that when $y=\frac{-za+\pi_{1}+\lambda \eta p+c_{2}-c_{1}}{\theta s+\lambda \eta p}$, there is F(x)≡0. At this time, any value of *x* is the stable strategy of the government departments, which means that the policy choice of the government departments will not change over time. When $y<\frac{-za+\pi_{1}+\lambda \eta p+c_{2}-c_{1}}{\theta s+\lambda \eta p}$, we have ${\left.\frac{dF(x)}{dx}\right|}_{x=1}<0$. At this time, *x* = 1 is the equilibrium point. When $y>\frac{-za+\pi_{1}+\lambda \eta p+c_{2}-c_{1}}{\theta s+\lambda \eta p}$, we have ${\left.\frac{dF(x)}{dx}\right|}_{x=0}<0$. At this time, *x* = 0 is the equilibrium point. Private pension institutions’ strategy remains stable when $z=\frac{x(\theta s+\lambda \Delta \pi +\lambda \eta p)-\Delta \pi +c_{4}+c_{7}-c_{3}-c_{6}}{c_{7}-c_{6}}$. Similarly, *y* = 1 is a stable point if $z>\frac{x(\theta s+\lambda \Delta \pi +\lambda \eta p)-\Delta \pi +c_{4}+c_{7}-c_{3}-c_{6}}{c_{7}-c_{6}}$, whereas *y* = 0 is the stable point when $z<\frac{x(\theta s+\lambda \Delta \pi +\lambda \eta p)-\Delta \pi +c_{4}+c_{7}-c_{3}-c_{6}}{c_{7}-c_{6}}$. In the same way, if $x=\frac{-yc_{8}-\pi_{3}+c_{5}+c_{8}}{a}$, then F(z)≡0, and the elderly’s strategy is stable. Participation (*z* = 1) is the optimal strategy if $x>\frac{-yc_{8}-\pi_{3}+c_{5}+c_{8}}{a}$; otherwise, non-participation (*z* = 0) is the optimal strategy.

According to the above analysis on the tripartite evolution strategy of government departments, private pension institutions, and the elderly, we can obtain the three-dimensional dynamic system of the evolutionary game:

$$\left\{\begin{array}{l} F_{x}(x,y,z)=x(1-x)[y(-\theta s-\lambda \eta p)-za+\pi_{1}+\lambda \eta p+c_{2}-c_{1}]\\ F_{y}(x,y,z)=y(1-y)[x(\theta s+\lambda \Delta \pi +\lambda \eta p)+z(c_{6}-c_{7})-\Delta \pi +c_{4}+c_{7}-c_{3}-c_{6}]\\ F_{z}(x,y,z)=z(1-z)(xa+yc_{8}+\pi_{3}-c_{5}-c_{8}) \end{array}\right.$$

When the speed of policy adjustment of the three parties no longer changes, that is, $F_{x}\left(x,y,z\right)$=$F_{y}\left(x,y,z\right)$=$F_{z}\left(x,y,z\right)$ = 0, the system reaches a relatively stable state. At this time, it is obtained that the game system has the following nine strategy equilibrium points: $E_{1}\left(0,0,0\right)$, $E_{2}\left(0,0,1\right)$, $E_{3}\left(0,1,0\right)$, $E_{4}\left(0,1,1\right)$, $E_{5}\left(1,0,0\right)$, $E_{6}\left(1,0,1\right)$, $E_{7}\left(1,1,0\right)$, $E_{8}\left(1,1,1\right)$, and $E_{9}\left(x^{*},y^{*},z^{*}\right)$. However, $E_{9}\left(x^{*},y^{*},z^{*}\right)$ is a mixed strategy point, which is generally not an ESS [34]. Therefore, we only consider the eight pure strategic points from $E_{1}$ to $E_{8}$. According to the Lyapunov stability theory [35], when the eigenvalues of the Jacobian matrix of the system are all negative, the system can be considered stable at this time. If at least one eigenvalue is positive, the system is unstable. The Jacobian matrix of the system is as follows:

$$J=\left[\begin{array}{ccc} (1-2x)\left[\begin{array}{c} y(-\theta s-\lambda \eta p)-za\\ +\pi_{1}+\lambda \eta p\\ +c_{2}-c_{1} \end{array}\right] & x(1-x)(-\theta s-\lambda \eta p) & x(1-x)(-a)\\ y(1-y)(\theta s+\lambda \Delta \pi +\lambda \eta p) & (1-2y)\left[\begin{array}{c} x(\theta s+\lambda \Delta \pi +\lambda \eta p)\\ +z(c_{6}-c_{7})-\Delta \pi +c_{4}\\ +c_{7}-c_{3}-c_{6} \end{array}\right] & y(1-y)(c_{6}-c_{7})\\ z(1-z)a & z(1-z)c_{8} & (1-2z)\left[\begin{array}{c} xa+yc_{8}+\pi_{3}\\ -c_{5}-c_{8} \end{array}\right] \end{array}\right]$$

Substitute the above eight equilibrium solutions into the Jacobian matrix, and calculate the corresponding eigenvalues. The specific results are shown in the Table 3 below.

From the parameter settings in the previous section, it is clear that the parameters involved in this model are non-negative and have $\pi_{1}>c_{1}$, $\pi_{3}>c_{5}$ and $c_{3}>c_{4}$. It can be seen from Table 3 that $E_{1}\left(0,0,0\right)$, $\;E_{3}\left(0,1,0\right)$, $E_{4}\left(0,1,1\right)$, and $E_{7}\left(1,1,0\right)$ all have non-negative eigenvalues ($\pi_{1}+\lambda \eta p+c_{2}-c_{1}>0$, $\pi_{3}-c_{5}>0$, $\Delta \pi -c_{4}+c_{3}>0$ and $a+\pi_{3}-c_{5}>0$), which do not meet the Lyapunov stability conditions, so they can be directly screened out. Therefore, we only need to analyze the stability of $E_{2}\left(0,0,1\right)$, $E_{5}\left(1,0,0\right)$, $E_{6}\left(1,0,1\right)$ and $E_{8}\left(1,1,1\right)$, and then obtain the following propositions:

**Proposition** **1.** *When*$-a+\pi_{1}+\lambda \eta p+c_{2}-c_{1}<0$*,*$\Delta \pi -c_{4}+c_{3}>0$*and*$-\pi_{3}+c_{5}+c_{8}<0$*,*$E_{2}\left(0,0,1\right)$*is the ESS. At this moment, government departments choose not to regulate, private pension institutions choose illegal operation, and the elderly choose to participate*.

**Proposition** **2.** *When* $\pi_{1}+\lambda \eta p+c_{2}-c_{1}>0$ *,* $\theta s+(\lambda -1)\Delta \pi +\lambda \eta p+c_{4}+c_{7}-c_{3}-c_{6}<0$ *and* $a+\pi_{3}-c_{5}-c_{8}<0$ *,* $E_{5}\left(1,0,0\right)$ *is the ESS. Under such conditions, government departments choose regulation, private pension institutions choose illegal operation, and the elderly choose not to participate.*

**Proposition** **3.** *When* $a-\pi_{1}-\lambda \eta p-c_{2}+c_{1}<0$ *,* $\theta s+(\lambda -1)\Delta \pi +\lambda \eta p+c_{4}-c_{3}<0$ *and* $-a-\pi_{3}+c_{5}+c_{8}<0$ *,* $E_{6}\left(1,0,1\right)$ *is the ESS. At this moment, government departments choose regulation, private pension institutions choose illegal operation, and the elderly choose participation.*

**Proposition** **4.** *When* $\theta s+a-\pi_{1}-c_{2}+c_{1}<0$ *,* $-\theta s-(\lambda -1)\Delta \pi -\lambda \eta p-c_{4}+c_{3}<0$ *and* $-a-\pi_{3}+c_{5}<0$ *,* $E_{8}\left(1,1,1\right)$ *is the ESS. Under such conditions, government departments choose regulation, private pension institutions choose standardized operation, and the elderly choose participation.*

## 4. Numerical Simulation Analysis of the Tripartite Evolutionary Game

In order to verify the stability of the four equilibrium points, $E_{2}\left(0,0,1\right)$, $E_{5}\left(1,0,0\right)$, $E_{6}\left(1,0,1\right)$, and $E_{8}\left(1,1,1\right)$ obtained from the theoretical point of view above, and further explore the impact of the initial strategy values and key parameters of game players on the stability of the system evolution, Matlab software was used for relevant numerical simulation analysis.

### 4.1. Numerical Simulation Analysis of Four Propositions

**Figure 1:**
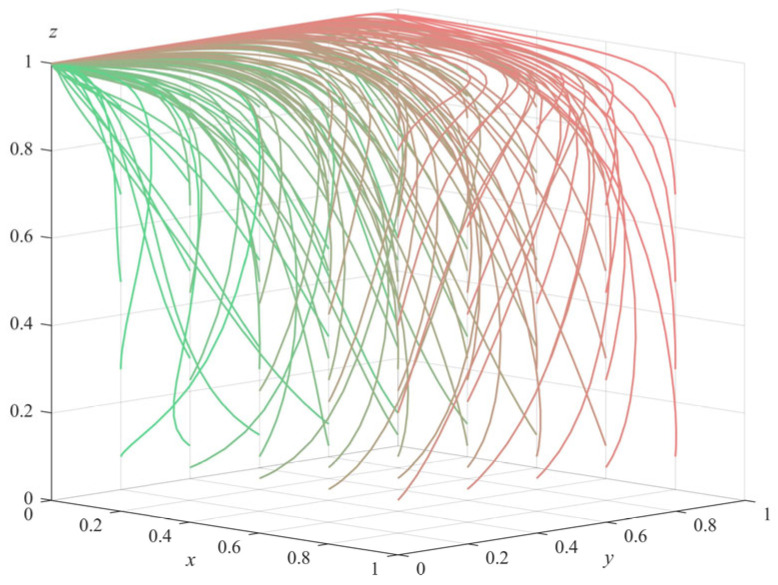
Simulation results of Proposition 1.

(1) Numerical simulation of Proposition 1: Under the condition that $-a+\pi_{1}+\lambda \eta p+c_{2}-c_{1}<0$, $\Delta \pi -c_{4}+c_{3}>0$, and $-\pi_{3}+c_{5}+c_{8}<0$ are satisfied, we set the parameters of the model as follows: $\pi_{1}\;$= 10, $\pi_{3}\;$= 12, Δπ = 2, $c_{1}\;$= 9, $c_{2}\;$= 2, $c_{3\;}$= 7, $c_{4}\;$= 5, $c_{5}\;$= 4, $c_{6}\;$= 3, $c_{7}\;$= 2, $c_{8}$ = 6, λ = 0.5, θ = 0.8, η = 0.8, *s* = 10, *p* = 10, *a* = 8. In order to reflect the evolution path of each agent more objectively and accurately, the simulation was randomly started from different initial strategies of the agents, the above parameters were combined, and it evolved 50 times with the system time. The final evolution result is shown in Figure 1 (The gradient of the line color from green to red indicates the evolution path of the initial value of the evolution strategy from (0.1,0.1,0.1) to (1,1,1)). It can be seen from the figure that the system eventually evolved to the equilibrium point $E_{2}\left(0,0,1\right)$; that is, the government departments choose not to supervise, the private pension institutions choose illegal operation, and the elderly choose participation. According to the conditions to be met in Proposition 1, $\pi_{1}+\lambda \eta p-c_{1}-a<-c_{2}$ means that the difference between the total revenue (including regulatory revenue and penalty revenue) and costs (including regulatory costs and subsidies to the elderly) of the government departments’ choice of regulatory strategies is less than the total loss caused by non-regulation; that is, the total revenue of the government departments’ choice of regulation is less than the total revenue of non-regulation. $\Delta \pi -c_{4}>-c_{3}$ means that the difference between the additional income and the operating cost of the private pension institutions’ choice of illegal operation is greater than the cost of the standardized operation; that is, the total income of the private pension institutions’ choice of illegal operation is greater than the total income of the standardized operation. $\pi_{3}-c_{5}-c_{8}>0$ means that the difference between the income and cost (including service fees and benefit losses) of the elderly who choose to participate is higher than the income of non-participation.

It can be seen that all parties have finally chosen the strategy of higher income in order to maximize their own interests. At the same time, the numerical simulation results of Proposition 1 also confirm the stability of $E_{2}$. However, this stable situation exposes the social phenomenon that the government departments have a weak sense of supervision, and the private pension institutions have a serious sense of profit seeking. In this case, in order to reduce the risks faced by the elderly in the process of enjoying care services, government departments should quickly reduce the cost of supervision and accelerate the change from a non-supervised state to a supervised state. At the same time, they should further increase the crackdown on the illegal operation of private pension institutions, so as to effectively restrain private pension institutions from committing illegal acts and constitute a certain deterrent effect on them.

**Figure 2:**
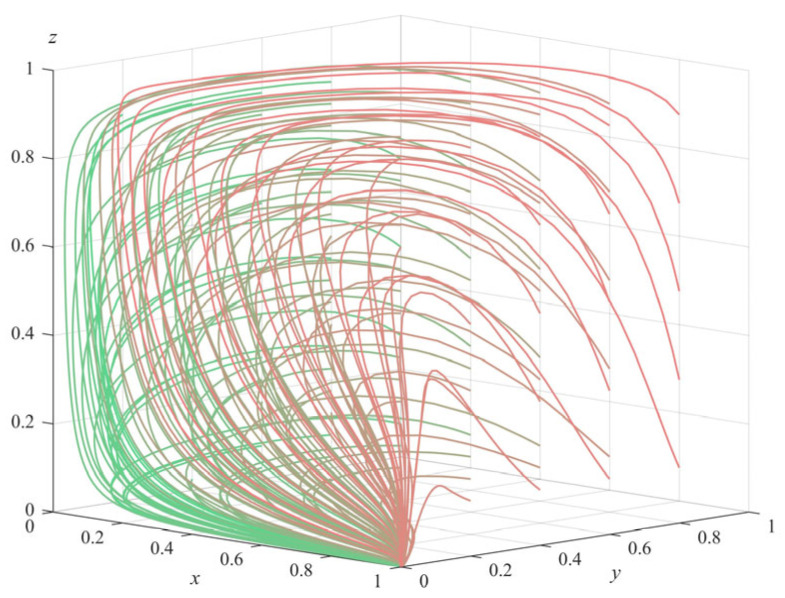
Simulation results of Proposition 2.

**Figure 3:**
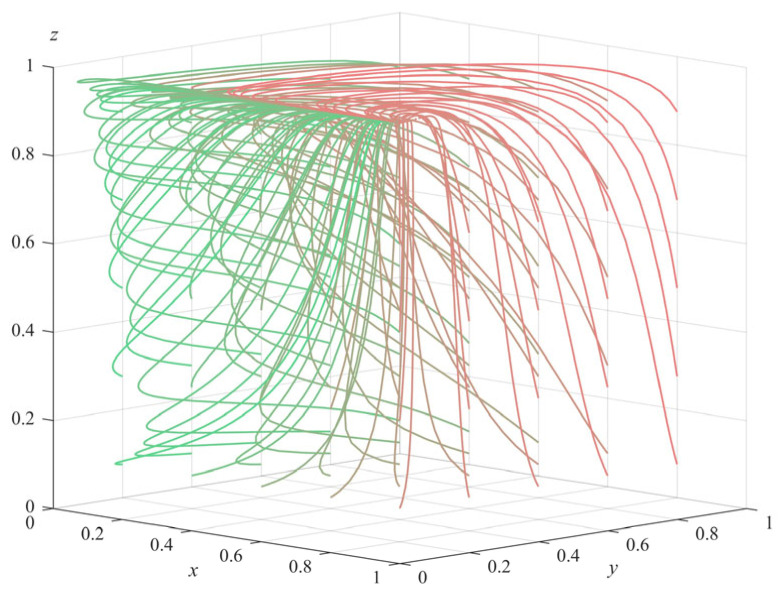
Simulation results of Proposition 3.

**Figure 4:**
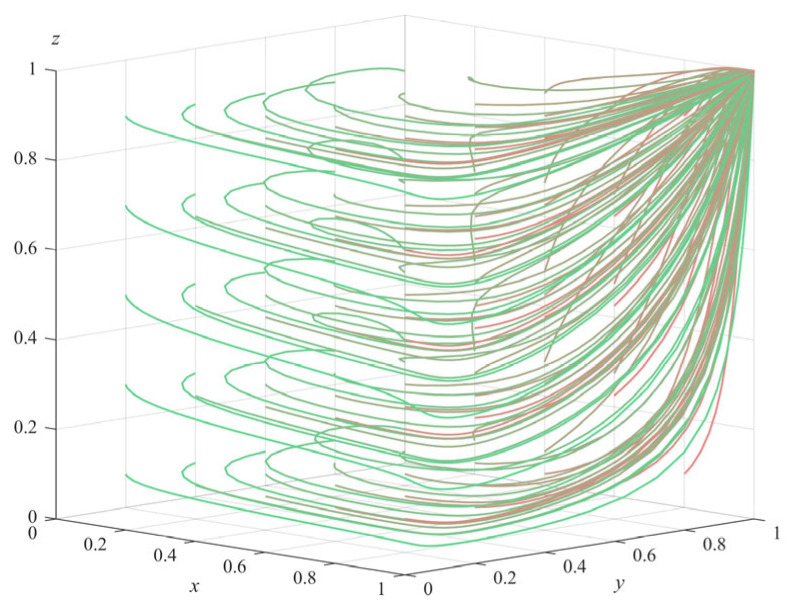
Simulation results of Proposition 4.

(2) Numerical simulation of Proposition 2: To satisfy the three inequality conditions of Proposition 2 ($\pi_{1}+\lambda \eta p+c_{2}-c_{1}>0$,$\theta s+(\lambda -1)\Delta \pi +\lambda \eta p+c_{4}+c_{7}-c_{3}-c_{6}<0$ and $a+\pi_{3}-c_{5}-c_{8}<0$), some parameters of the model were adjusted to Δπ = 20 and $c_{8}$ = 18, and the rest of the parameter values remained the same as the initial settings of the numerical simulation of Proposition 1. The simulation also started randomly from different strategy combinations for each subject and evolved 50 times with the system time; the results obtained are shown in Figure 2 (The gradient of the line color from green to red indicates the evolution path of the initial value of the evolution strategy from (0.1,0.1,0.1) to (1,1,1)). It can be seen from the figure that the system eventually evolved to the equilibrium point $E_{5}$(1,0,0); that is, government departments choose regulation, private pension institutions choose illegal operation, and the elderly choose non-participation. Further analysis of the three conditions satisfied by Proposition 2 shows that $\pi_{1}+\lambda \eta p-c_{1}>-c_{2}$ means that the benefit of the government departments’ choosing to regulate is higher than that of not regulating; $\theta s-c_{3}-c_{6}<(1-\lambda )\Delta \pi -\lambda \eta p-c_{4}-c_{7}$ means that the benefit of private pension institutions’ choosing standardized operation is smaller than that of violating; $a+\pi_{3}-c_{5}-c_{8}<0$ means that the benefit of the elderly’s choosing participation is smaller than that of non-participation. Therefore, it can be inferred that the final strategy choices of each subject are related to the maximization of their own interests, and the numerical simulation results of Proposition 2 are consistent with the theoretical derivation results. This situation corresponds to the stage where government departments have regulatory awareness, but the regulatory methods and mechanisms are not mature enough. At this time, the government departments should fully invest in the supervision, improve the success rate of supervision as much as possible, and severely crack down on the illegal operation of private pension institutions. At the same time, the government departments can set more incentive subsidy policies to promote the private pension institutions to change to the standard operation direction.
(3) Numerical simulation of Proposition 3: Under the condition that $a-\pi_{1}-\lambda \eta p-c_{2}+c_{1}<0$,$\theta s+(\lambda -1)\Delta \pi +\lambda \eta p+c_{4}-c_{3}<0$ and $-a-\pi_{3}+c_{5}+c_{8}<0$, some parameters of the model were adjusted: Δπ = 21, and $c_{1}\;$= 6, while other parameter values remain the same as those set in the numerical simulation of Proposition 1. Again evolving 50 times with the system time, the final simulation results obtained are shown in Figure 3 (The gradient of the line color from green to red indicates the evolution path of the initial value of the evolution strategy from (0.1,0.1,0.1) to (1,1,1)). It can be seen from Figure 3 that the system eventually evolves to equilibrium point $E_{6}\left(1,0,1\right)$. Similarly, by analyzing the three prerequisites of Proposition 3, we can see that the three parties finally choose their own strategies with higher benefits, and the simulation results of Proposition 3 also verified the stability of $E_{6}\left(1,0,1\right)$. This situation shows that the regulatory system of government departments is not perfect enough, and private pension institutions are too strong in their profit-seeking behavior, in which it is very easy to infringe on the legitimate rights and interests of the elderly. In this case, the government departments should further strengthen the supervision, try to find and cut off the channels for illegal operation and profit of private pension institutions, further improve the subsidy and punishment policies for private pension institutions, promote their evolution towards standardized operation, and effectively protect the legitimate rights and interests of the elderly.
(4) Numerical simulation of Proposition 4: In order to meet the three conditions of Proposition 4 ($\theta s+a-\pi_{1}-c_{2}+c_{1}<0$,$-\theta s-(\lambda -1)\Delta \pi -\lambda \eta p-c_{4}+c_{3}<0$ and $-a-\pi_{3}+c_{5}<0$) some parameters of the model were adjusted to $\pi_{3}$ = 8, Δπ = 10, $c_{1}$ = 2, and *a* = 1, while the other parameter values were consistent with the initial settings in the numerical simulation of Proposition 1. It also evolved 50 times with the system time, and the final simulation results are shown in Figure 4 (The gradient of the line color from green to red indicates the evolution path of the initial value of the evolution strategy from (0.1,0.1,0.1) to (1,1,1)). We can see that the system finally evolved to the ideal stable state of equilibrium point $E_{8}\left(1,1,1\right)$. Similarly, analyzing the three preconditions of Proposition 4, it is known that each subject will eventually choose the strategy with the largest gain, and the numerical simulation results verify the stability of $E_{8}\left(1,1,1\right)$. This stable situation corresponds to the mature stage of regulation and governance of senior care services. Government departments are able to reasonably adjust the cost of regulation and rationally formulate reward and punishment schemes suitable for private pension institutions and the elderly, which regulate the behavior of each subject and facilitate the long-term development of social senior care services.

### 4.2. Simulation Analysis of the Initial Strategy of Participating Subjects

**Figure 5:**
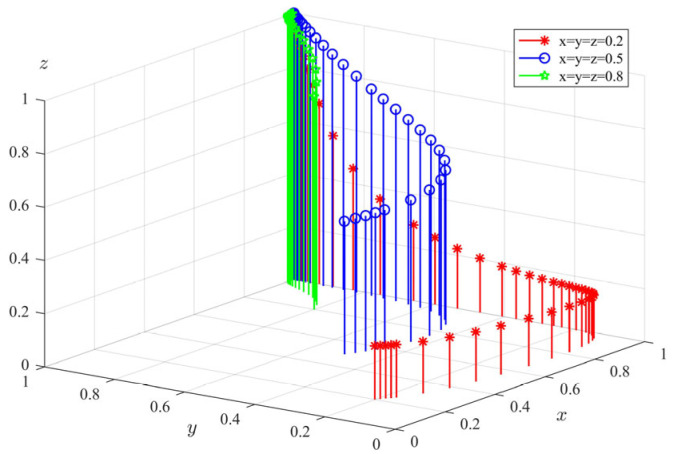
Simulation results of the initial strategy of participating subjects.

Taking Proposition 4 as an example, when $\theta s+a-\pi_{1}-c_{2}+c_{1}<0$, $-\theta s-(\lambda -1)\Delta \pi -\lambda \eta p-c_{4}+c_{3}<0$ and $-a-\pi_{3}+c_{5}<0$, we set the model parameter values as follows: $\pi_{1\;}$= 10, $\pi_{3}\;$= 8, Δπ  = 10, $c_{1}\;$= 2, $c_{2}\;$= 2, $c_{3}\;$= 7, $c_{4}\;$= 5, $c_{5}\;$= 4, $c_{6}\;$= 3, $c_{7}\;$= 2, $c_{8\;}$= 6, λ = 0.5, θ = 0.8, η = 0.8, *s* = 10, *p* = 10, *a* = 1. The initial strategies of government departments, private pension institutions, and the elderly were also set as (*x, y, z*) = (0.2,0.2,0.2), (*x, y, z*) = (0.5,0.5,0,5), and (*x, y, z*) = (0.8,0.8,0.8). The dynamic evolution process of each subject obtained by substituting the three initial strategy combination values into the model for simulation is shown in Figure 5. It can be seen from Figure 5 that in this case, the policy choices of government departments, private pension institutions, and the elderly eventually evolve to “supervision,” “standardized operation,” and “participation” strategies, which indicates that the final evolution results of the system have no inevitable relationship with the initial strategy values of each subject, and the size of the initial strategy values will only affect the speed of the evolution of each subject to a stable state. It can be seen from the figure that with the increase in the initial strategy value, private pension institutions and the elderly will reach a stable state faster, while the government departments will do the opposite. Considering the reality, it is because with the increasing probability of private pension institutions operating in a regulated manner, the probability of the elderly experiencing infringement of their rights and interests when they choose to participate in senior care services will become smaller and smaller, and government departments will choose to slow down the speed of regulation at this time. However, when the probability of standardized operation of the private pension institutions is low, the government departments will quickly evolve to a comprehensive regulatory state in a short time to protect the rights and interests of the elderly.

### 4.3. Simulation Analysis of External Parameters

The stability condition ($-c_{2}<\pi_{1}-\theta s-a-c_{1}$, $(1-\lambda )\Delta \pi -\lambda \eta p-c_{4}<\theta s-c_{3}$, $\pi_{3}+a-c_{5}>0$) of the ideal stable point $E_{8}\left(1,1,1\right)$ in Proposition 4 shows that only when the government departments choose the regulatory strategy with higher returns than the non-regulatory strategy, private pension institutions choose the standardized operation with higher returns than the illegal operation, and the elderly choose the participation strategy with higher returns than the non- participation strategy, the system will gradually evolve to the stable state of {regulation, standardized operation, participation}. Therefore, Proposition 4 was taken as an example to further explore the impact of external parameters on the evolutionary behavior of each subject by combining this situation with parameter factors. The initial values of the external parameter simulation analysis were kept consistent with the numerical simulation of Proposition 4, while the initial strategy of each subject was taken as (*x, y, z*) = (0.5,0.5,0,5) to ensure the objective accuracy of the simulation results.

(1) The impact of regulatory success rate (*λ*) on tripartite evolutionary behavior

**Figure 6:**
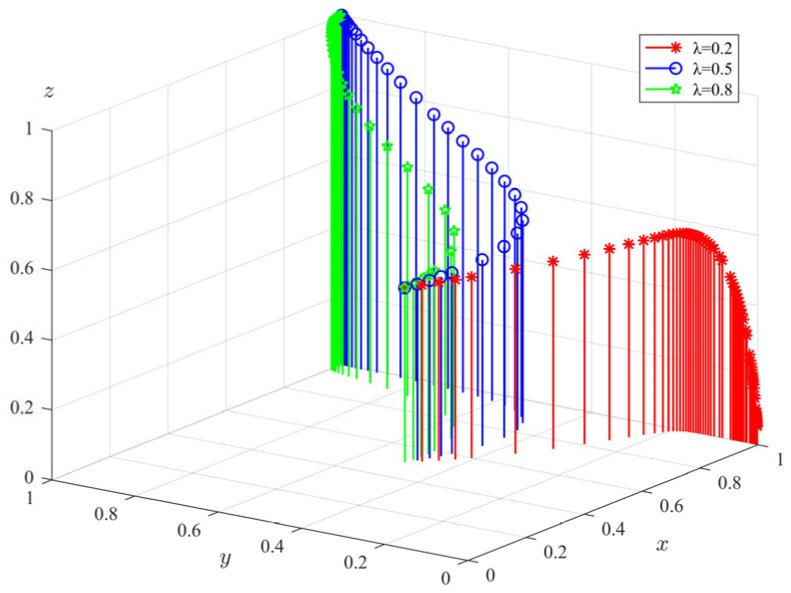
The impact of regulatory success rate (*λ*) on the evolution of tripartite behavior.

Keeping the other parameters of the model unchanged and changing the regulatory success rate of government departments, taking *λ* = 0.2, 0.5, and 0.8, the evolutionary path of each subject was obtained as in Figure 6. It can be seen from the figure that when the regulatory success rate is too low, private pension institutions will gradually evolve to illegal operation, and the participation rate of the elderly will gradually approach zero. With the increase in *λ*, the rate of private pension institutions’ evolving to fully standardized operation has also accelerated, and the elderly have also accelerated to fully participate in the state. However, the rate at which government departments evolve to full regulation is inversely proportional to the rate of regulatory success. When the regulatory success rate is high, the government departments will slow down the supervision rate, because private pension institutions will soon evolve to a fully standardized operating state. Conversely, when regulatory success rate is low, and private pension institutions tend to choose to operate in violation, government departments will quickly evolve to a fully regulated state. Obviously, the success rate of regulation will have a significant effect on the behavior decision making of private pension institutions. If the government can accurately detect and severely punish the illegal operation of some pension institutions every time in the supervision work, other private pension institutions in the same industry will also take a warning and gradually regulate their own operation. In the case that private elderly care institutions comply with industry norms and legally provide elderly care services, the elderly will also choose participation strategies. On the contrary, if the government’s supervision is weak, so that some private pension institutions can continue to make illegal profits, all pension institutions will ignore the law for the benefit in the long run. In this case, the elderly will also choose not to participate because of improper operation of the elderly care institutions.

(2) The impact of subsidy coefficient (*θ*) on tripartite evolutionary behavior

**Figure 7:**
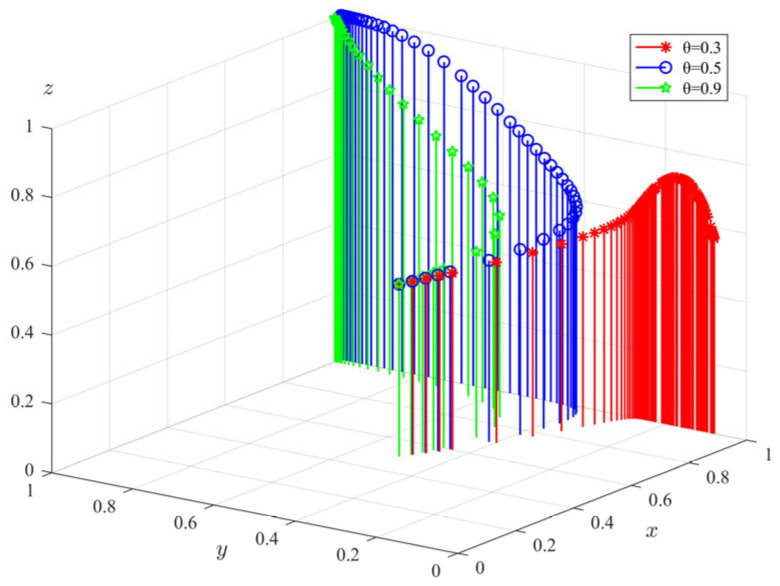
The impact of subsidy coefficient (*θ*) on the evolution of tripartite behavior.

Keeping other parameters of the model unchanged, we changed the subsidy coefficient of government departments and took *θ* = 0.3, 0.5, 0.9; the evolution path of each subject is shown in Figure 7. It can be seen from the figure that when the subsidy coefficient is small, private pension institutions tend to choose illegal operation, and the elderly tend not to participate. With the increase in *θ,* the probability of standardized operation of private elderly care institutions and the elderly’s choice to participate will increase, and the rate of both stabilizing to the ideal state is in direct proportion to the *θ*. However, the evolution rate of government departments’ choice of regulation decreases with the increase in the subsidy coefficient. When the subsidy coefficient is too large, the stable supervision rate of government departments does not reach the ideal state of 100%. This shows that a too high subsidy coefficient will reduce the regulatory willingness of government departments. It can be seen that increasing the subsidies of the government departments to the elderly care institutions can significantly promote their transformation to standardized business behavior. Private pension institutions are essentially profitable enterprises, and the government’s support for their operation is very important. If the pension institution can earn more profit under the subsidy of the government department than its income under the illegal operation, it must choose to accept the subsidy and regulate the operation. However, if the subsidies are too small and the revenue of private pension institutions is low, they will choose illegal operation to seek more benefits. However, for the government departments, the funds that can be allocated to the supervision of elderly care services are limited. If the subsidies given to the elderly care institutions are too high, this will greatly reduce the government’s supervision probability. The behavior decision of the elderly will be directly affected by the behavior of private pension institutions.

(3) The impact of penalty coefficient (*η*) on tripartite evolutionary behavior

**Figure 8:**
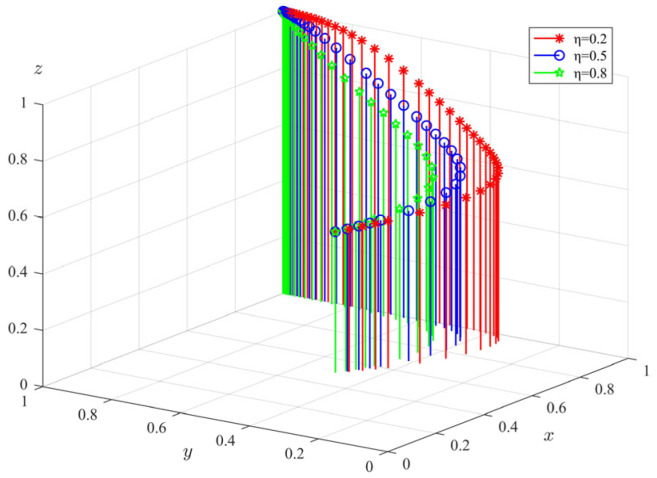
The impact of penalty coefficient (*η*) on the evolution of tripartite behavior.

Keeping other parameters of the model unchanged, we changed the penalty coefficient of government departments and took *η* = 0.2, 0.5, 0.8; the evolution path of each subject is shown in Figure 8. The graph shows that an increase in the penalty coefficient causes a slight acceleration in the rate at which government departments choose to evolve their regulatory behavior to a steady state, but the overall change is not significant. The evolutionary rate of private pension institutions’ choosing to regulate their operations and the elderly’s choosing to participate is significantly accelerated with the increase in *η*. The above results show that the increase in the punishment of government departments can effectively accelerate the evolution of private pension institutions to a standardized operating state and can also improve the participation of the elderly in using elderly care services. It is easy to understand that when the government imposes heavy penalties on the elderly care institutions for violating the regulations, the fine is a great loss to the institutions. Any enterprise is afraid of loss, because it will damage the overall interests of the enterprise. In order to ensure that the private pension institutions can continue to operate in the future, they will gradually regulate their own operation behavior and dare not know the law. The greater the punishment, the higher the loss to the elderly care institutions, and the better the effect of promoting their standardized operation. For the government department, the fine is a sum of income, which gives the government department more power to supervise. The willingness of the elderly to participate is directly proportional to the probability of standardized operation of private pension institutions. In other words, the increase in penalty is also conducive to improving the trust of the elderly in private pension institutions.

(4) The impact of additional benefits (Δπ) on tripartite evolutionary behavior

**Figure 9:**
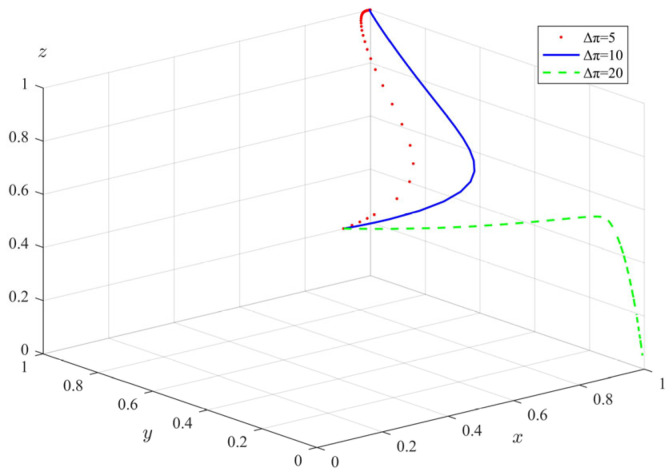
The impact of additional benefits (Δπ) on the evolution of tripartite behavior.

In the same way, we changed the additional benefits obtained by private pension institutions from illegal operations, and took Δπ = 5, 10, 20; the evolution path is shown in Figure 9. It can be seen that with the increase in Δπ, the rate of government departments’ evolving to the state of full supervision is significantly accelerated. When Δπ = 20, private pension institutions have evolved from standardized operation to illegal operation, and the participation rate of the elderly has gradually evolved to zero. The above results show that under the condition that the supervision and rewards and punishments of government departments remain unchanged, when Δπ is small, private pension institutions will choose to operate in a standardized manner. As Δπ increases, the evolution rate of private pension institutions’ choosing regulated operation decreases significantly, and when Δπ is higher than a certain threshold, private pension institutions will eventually choose illegal operation. In this case, the elderly will also choose not to participate due to risk aversion. Since the nature of private pension institutions is profitable, the higher additional income for them is indeed very attractive. If the subsidy and punishment of the government departments are not large enough, private pension institutions will violate the moral and legal bottom line in order to pursue money. In this case, in order to protect the elderly who are using pension services, the government departments will also rapidly evolve to a comprehensive regulatory state. For a long time, the elderly will also gradually withdraw, due to the illegal operation of pension institutions.

(5) The impact of fixed subsidies (*a*) on tripartite evolutionary behavior

**Figure 10:**
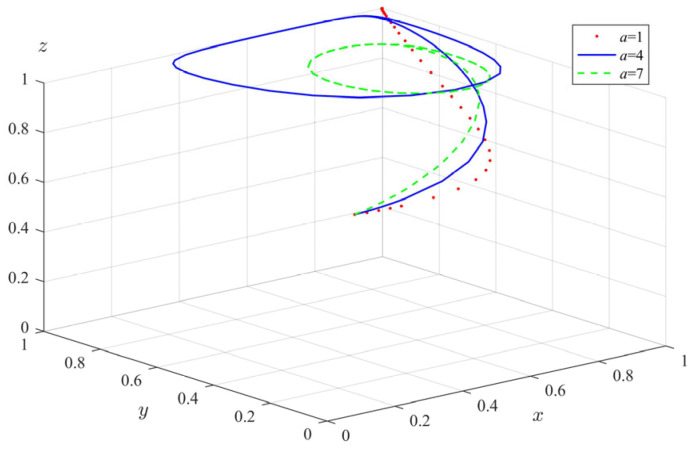
The impact of fixed subsidies (*a*) on the evolution of tripartite behavior.

Similarly, we changed the fixed subsidy value given by the government departments to the elderly to participate in the elderly care service and took *a* = 1, 4, 7. The evolution path of each subject is shown in Figure 10. It can be seen that with the increase in the government departments’ fixed subsidies for the elderly, the rate of the elderly’s evolution to participation behavior is significantly accelerated. However, with the increase in *a*, the regulatory behavior of government departments will fluctuate periodically, and the rate of fluctuation is proportional to *a*. The probability of standardized operation of private pension institutions also fluctuates with the increase in *a*. The above results show that the increase in fixed subsidies for the elderly can effectively improve the enthusiasm and participation of elderly groups in using elderly care services. However, government departments may have regulatory fluctuations due to the high subsidy value and may not take 100% regulatory measures. In this case, some private pension institutions will also tend to choose illegal operation, because the government departments have not implemented comprehensive supervision, which may bring higher risks to the elderly involved in elderly care. In fact, for the elderly, the greater the government subsidies, the stronger their willingness to participate in the elderly care service. However, for the government departments, the funds for pension services are limited. If the fixed subsidies allocated to the elderly are too high, it will greatly reduce the regulatory will. Once the government departments relax their supervision, private pension institutions will want to take illegal means to make profits. Therefore, government departments must set a reasonable subsidy value to promote the smooth operation of pension service supervision.

(6) The impact of regulatory fixed costs ($c_{1}$) on tripartite evolutionary behavior

**Figure 11:**
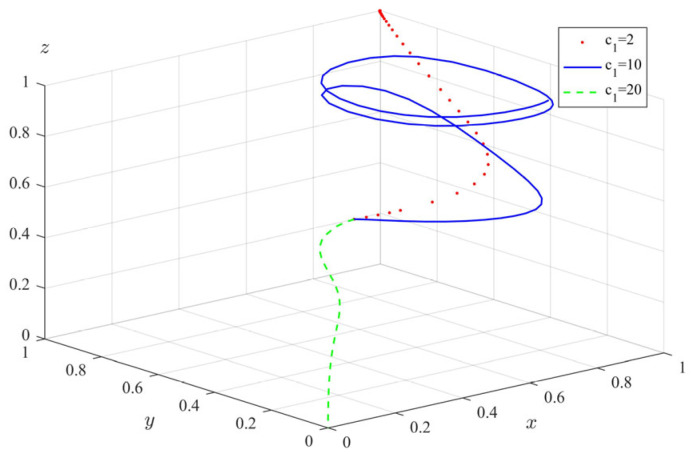
The impact of regulatory fixed costs ($c_{1}$) on the evolution of tripartite behavior.

Finally, we explored the impact of regulatory fixed costs on the tripartite evolution strategy. We changed the fixed cost of the government departments’ implementation of supervision, taking $c_{1}\;$= 2, 10, 20, and obtained the evolution path of each subject as shown in Figure 11. It can be seen that when $c_{1}\;$= 2, government departments will eventually evolve to full supervision, private pension institutions will evolve to fully standardized operation, and the elderly will evolve to full participation. When $c_{1}\;$= 10, the behavior evolution of government departments and private pension institutions fluctuates periodically, and the rate of the elderly’s evolving to a stable state slows down significantly. When $c_{1}\;$= 20, due to the high cost of supervision, the government departments finally choose not to supervise. The private pension institutions evolve into illegal operation due to the lack of supervision by the government. The elderly also choose non-participation in the illegal operation of private pension institutions. The above shows that as $c_{1}$ increases, the regulatory behavior of government departments will show cyclical fluctuations, and when $c_{1}$ exceeds a certain threshold, government departments will choose not to regulate because the cost is too high. The behavior evolution results of private pension institutions and the elderly are closely related to the supervision rate of government departments. Obviously, the government’s own supervision requires a certain amount of human, material, and financial costs. If the total regulatory cost is too high, it is difficult for the government departments to have the motivation to continue to implement the regulatory strategy. Once the government department has not fully supervised, private pension institutions want to take advantage of the opportunity to make profits, and the elderly may also be injured. Therefore, in order to reduce the probability of illegal operation of private pension institutions, the government departments must take effective measures to reduce the cost of supervision, so as to ensure a higher regulatory will to achieve the purpose of protecting the elderly.

## 5. Discussion

He and Yue et al. have built a two-party evolutionary game model between the government and the pension institutions for the government’s supervision of the elderly care institutions [1,18]. Through simulation experiments, they explored the impact of different initial conditions and changes in parameters such as punishment and operating subsidies of government on the evolution process and results and then put forward corresponding policy recommendations for government decision making. However, they all ignored that the behavior decision of the elderly group in the elderly care service supervision system will also have a great impact on the behavior decisions of the government and private elderly care institutions. Therefore, this paper brings the elderly group into the evolutionary game model and, under the social background of frequent illegal operation of private elderly care institutions, further explores how the government departments should regulate the operation of private pension institutions, protect the legitimate rights and interests of the elderly group, and achieve the long-term goal of promoting the healthy development of elderly care services. In addition, the construction of the evolutionary game model that includes government departments, private pension institutions, and the elderly has also enriched the research of the game model in the field of pension service supervision.

This paper mainly studies the behavior decisions and results of government departments, private pension institutions, and the elderly in the process of pension service supervision. The parameters such as supervision cost, subsidy, and penalty coefficients will affect the supervision decisions of government departments. The behavior decisions of private pension institutions are often affected by the regulatory policies of government departments, additional benefits, and other factors. The regulatory policies and fixed subsidies of the government departments will also have a significant impact on the behavior decision making of the elderly. In addition, in the long-term decision-making process, the behavior decisions of the three subjects will also interact. Therefore, this paper provides insights into how government departments, private pension institutions, and the elderly make behavioral decisions under different propositions, different initial strategic values, and different model parameters. The following three points summarize the main conclusions of this paper.

(1) According to simulation analysis, there are four ESSs in the pension service supervision system, namely $E_{2}\left(0,0,1\right),E_{5}\left(1,0,0\right),\;E_{6}\left(1,0,1\right),\;\mathrm{and}\;E_{8}\left(1,1,1\right)$. The final result of system evolution depends on the relative return value of each agent’s selection strategy. Revenue is the decisive factor that affects the evolution of the game players’ strategies. For the government department, only when the net income of implementing the regulatory strategy is positive, will the government department implement the regulatory strategy; otherwise, it will choose the non-regulatory strategy. If the government department adopts the non-regulatory strategy, the private pension institutions are likely to choose the illegal operation strategy, in which it is very easy to cause the rights of the elderly to be violated. When the government department chooses to implement the regulatory strategy and the net income of private pension institutions’ choosing to regulate operation is positive, private pension institutions will choose to regulate operation, and the elderly will also choose to participate. In the four ESSs, $E_{8}\left(1,1,1\right)$ indicates that the ideal stable state is of government departments’ choosing supervision, private pension institutions’ choosing standardized operation, and the elderly’s choosing participation strategy. It also corresponds to the mature stage of pension service supervision and governance, which is conducive to the long-term development of social pension service. The other three ESSs all represent the governance stage in which private pension institutions have a too strong profit-seeking behavior, and the regulatory system of government departments is not perfect. In these three cases, the government departments need to work hard to improve the regulatory efficiency and formulate reasonable regulatory policies according to the actual situation to promote the evolution of the system to an ideal stable state.
(2) No matter what the initial strategy of the participants is, they can change their behavior through observation, learning, and imitation, and finally evolve to the optimal stable strategy (under the condition of proposition 4): namely, the government departments choose to supervise, the private pension institutions choose to regulate the operation, and the elderly choose to participate. This shows that the final evolution result of the system is not necessarily related to the initial strategy value of each agent, but the size of the initial strategy value will affect the rate of each agent’s evolution to a stable state. The smaller the probability that private pension institutions initially choose to operate in a standardized manner, the greater the rate at which government departments evolve to complete supervision. This is because in this case, the probability of the elderly being infringed upon by institutions is very high. The government departments need to protect the legal rights and interests of the elderly, so they need to complete the comprehensive supervision of the elderly care institutions in a short time. The rate at which the elderly evolve to full participation is positively correlated with the rate at which private pension institutions evolve to standardized operation. This shows that the elderly also have a sense of seeking benefits and avoiding disadvantages. Only when the operation of the elderly care institutions is safe and standardized enough, will they have a stronger willingness to participate in the use of elderly care services.
(3) The increase in the success rate of government supervision, subsidy coefficient and punishment coefficient, or the reduction of its own supervision cost and fixed subsidies for the elderly can increase the probability of private pension institutions’ evolving towards standardized operation. In addition, we need to pay attention to the impact of additional income on the behavior evolution of private pension institutions. If the extra income is large enough, private pension institutions will choose to operate in violation of regulations, which will do great harm to the elderly. Therefore, in order to maintain the good order of the elderly care service market, the government departments must crack down on the illegal profit-making behavior of private elderly care institutions and cut off their profit-making channels as much as possible.

For the government department, if the evolution result is not the ideal result, the government can take measures to promote the evolution of the game to the ideal result, for example, increase the punishment for illegal operation and the subsidy for standardized operation. These measures can directly affect private pension institutions and effectively promote private pension institutions to choose standardized operation. In addition, by improving the success rate of supervision, reducing the cost of supervision, and providing fixed subsidies to the elderly, the supervision probability of government departments can also be directly improved. These measures play a key role in reducing the occurrence of illegal business events.

## 6. Conclusions

The pension service supervision system is a relatively complex system, which is composed of government departments, private pension institutions, and the elderly and other stakeholders. How to strengthen the supervision of government departments on elderly care services and improve the awareness of private elderly care institutions’ standardized operation has been the focus of the elderly care field. However, most of the existing articles are based on the game analysis between the government and the pension institutions, ignoring the synergy between multiple participants. Therefore, this paper used EGT to build a three-party evolutionary game model including government departments, private pension institutions, and the elderly, and used Matlab(2016b) software to conduct numerical simulations to verify the ESS of the system and further explore the impact of the initial strategy and external variables on the subjects’ behavior decision making. The evolutionary game model is conducive to identifying the decision-making behavior of government departments, private pension institutions, and the elderly. In addition, the model can not only help the government to formulate relevant regulatory measures, but also guide the elderly to make strategic choices in different scenarios and dynamic situations.

Based on the results of this study, we put forward some policy recommendations that are more applicable to developing countries, including China, to regulate the operation of private pension institutions and effectively protect the legitimate rights and interests of the elderly. The specific suggestions are as follows: Firstly, in order to reduce the occurrence of illegal operation of private pension institutions, the government departments should always maintain the regulatory status. Because private pension institutions are profit-oriented, if the government does not supervise them, they will not choose standardized management strategies. Government departments need to take corresponding measures to improve regulatory efficiency. Secondly, in order to reduce the possibility of illegal operation of private pension institutions, the government needs to increase the punishment of illegal operations. Larger punishment can effectively regulate the management decisions of pension institutions. At the same time, it is also possible to adopt a hierarchical incentive policy, that is, to give more subsidies to the elderly care institutions with a higher standard of business reputation and promote them to provide better services. Thirdly, government departments can enhance the safety awareness of the elderly through publicity and education and formulate appropriate incentive policies to guide the elderly to participate in the supervision of pension institutions. Finally, in view of the limited regulatory funds of the government and the increasing demand for elderly care services, the government departments also need to make long-term financial plans to reduce the regulatory costs as much as possible and ensure the long-term stable operation of the elderly care service market.

There are still some areas for improvement in this article. For example, the impact of the elderly on the regulatory role and reputation evaluation of pension institutions is not taken into account. In the future, these factors can be considered for inclusion in the model, which will allow for deeper model analysis. In addition, most articles in the field of operations management contain strong assumptions, and similar problems may arise in this article. These issues will be addressed in future studies.

## Figures and Tables

**Table 1:** Parameters and definitions.

Parameters	Descriptions
$\pi_{1}$	Total revenue when government departments choose $G_{1}$ strategy
$\pi_{2}$	Basic operating income of private pension institutions when choosing $P_{1}$ strategy
$\pi_{3}$	The benefits of using elderly care services when the elderly choose $E_{1}$ strategy
Δ*π*	Additional benefits obtained by private pension institutions by choosing $P_{2}$ strategy
$c_{1}$	Fixed costs invested by government departments when choosing the $G_{1}$ strategy
$c_{2}$	Total losses suffered by government departments when choosing $G_{2}$ strategy
$c_{3}$	The cost invested by private pension institutions when choosing $P_{1}$ strategy
$c_{4}$	The cost invested by private pension institutions when choosing $P_{2}$ strategy
$c_{5}$	Service fees paid by the elderly when they choose $E_{1}$ strategy
$c_{6}$	Private pension institutions choose $P_{1}$ strategy, but the elderly choose $E_{2}$ strategy, resulting in operational losses
$c_{7}$	Private pension institutions choose $P_{2}$ strategy, but the elderly choose $E_{2}$ strategy, resulting in operational losses
$c_{8}$	The benefit loss of the elderly caused by the choice of $P_{2}$ strategy by private pension institutions
*a*	Fixed subsidies given by government departments when the elderly choose $E_{1}$ strategy
*λ*	regulatory success rate of government departments
*s*	Subsidies from government departments when private pension institutions choose $P_{1}$ strategy
*θ*	Subsidy coefficient of government departments
*p*	Punishments of government departments when private pension institutions choose $P_{2}$ strategy
*η*	Penalty coefficient of government departments
**Variables**	**Descriptions**
*x*	Probability of government departments’ choosing $G_{1}$ strategy
*y*	Probability of private pension institutions’ choosing $P_{1}$ strategy
*z*	Probability of the elderly choosing $E_{1}$ strategy

**Table 2:** The payoff matrix. (The combined income matrix).

		Government Departments			
		$G_{1}$ Strategy (*x*)		$G_{2}$ Strategy (1−*x*)	
		The Elderly		The Elderly	
		$E_{1}$ Strategy (*z*)	$E_{2}$ Strategy (1−*z*)	$E_{1}$ Strategy (*z*)	$E_{2}$ Strategy (1−*z*)
**Private pension institutions**	$P_{1}$ **strategy (*y*)**	$\pi_{1}$ *−* $c_{1}$ *−θs−a* $\pi_{2}+$ *θs−* $c_{3}$ $\pi_{3}$ *−* $c_{5}+a$	$\pi_{1}$*−*$c_{1}$*−θs* $\pi_{2}+$*θs−*$c_{3}$*−*$c_{6}$ 0	*−* $c_{2}$ $\pi_{2}$ *−* $c_{3}$ $\pi_{3}$ *−* $c_{5}$	*−*$c_{2}$ $\pi_{2}$*−*$c_{3}$*−*$c_{6}$ 0
	$P_{2}$ **strategy (1−*y*)**	$\pi_{1}$ *−* $c_{1}+$ *ληp−a* $\pi_{2}+$ $(1-\lambda )\;\Delta \pi -\lambda \eta p-c_{4}$ $\pi_{3}$ *−* $c_{5}$ *−* $c_{8}+$ *a*	$\pi_{1}$*−*$c_{1}+$*ληp* $\pi_{2}+$$(1-\lambda )\;\Delta \pi -\lambda \eta p-c_{4}$*−*$c_{7}$ 0	*−* $c_{2}$ $\pi_{2}+$ $\Delta \pi -c_{4}$ $\pi_{3}$ *−* $c_{5}$ *−* $c_{8}$	*−*$c_{2}$ $\pi_{2}+$$\Delta \pi -c_{4}$*−*$c_{7}$ 0

**Table 3:** The eigenvalues of Jacobian matrix J.

**Equilibrium Points**	Eigenvalue 1	Eigenvalue 2	Eigenvalue 3
$E_{1}\left(0,0,0\right)$	$\pi_{1}+\lambda \eta p+c_{2}-c_{1}$	$-\Delta \pi +c_{4}+c_{7}-c_{3}-c_{6}$	$\pi_{3}-c_{5}-c_{8}$
$E_{2}\left(0,0,1\right)$	$-a+\pi_{1}+\lambda \eta p+c_{2}-c_{1}$	$-\Delta \pi +c_{4}-c_{3}$	$-\pi_{3}+c_{5}+c_{8}$
$E_{3}\left(0,1,0\right)$	$-\theta s+\pi_{1}+c_{2}-c_{1}$	$\Delta \pi -c_{4}-c_{7}+c_{3}+c_{6}$	$\;\pi_{3}-c_{5}$
$E_{4}\left(0,1,1\right)$	$-\theta s-a+\pi_{1}+c_{2}-c_{1}$	$\Delta \pi -c_{4}+c_{3}$	$-\pi_{3}+c_{5}$
$E_{5}\left(1,0,0\right)$	$-\pi_{1}-\lambda \eta p-c_{2}+c_{1}$	$\theta s+\left(\lambda -1\right)\Delta \pi +\lambda \eta p+c_{4}+c_{7}-c_{3}-c_{6}$	$\;a+\pi_{3}-c_{5}-c_{8}$
$E_{6}\left(1,0,1\right)$	$a-\pi_{1}-\lambda \eta p-c_{2}+c_{1}$	$\theta s+\left(\lambda -1\right)\Delta \pi +\lambda \eta p+c_{4}-c_{3}$	$-a-\pi_{3}+c_{5}+c_{8}$
$E_{7}\left(1,1,0\right)$	$\theta s-\pi_{1}-c_{2}+c_{1}$	$-\theta s-\left(\lambda -1\right)\Delta \pi -\lambda \eta p-c_{4}-c_{7}+c_{3}+c_{6}$	$a+\pi_{3}-c_{5}$
$E_{8}\left(1,1,1\right)$	$\theta s+a-\pi_{1}-c_{2}+c_{1}$	$-\theta s-\left(\lambda -1\right)\Delta \pi -\lambda \eta p-c_{4}+c_{3}$	$-a-\pi_{3}+c_{5}$

## Data Availability

The study did not report any data.
